# Diffusion tensor tractography of brainstem fibers and its application in pain

**DOI:** 10.1371/journal.pone.0213952

**Published:** 2020-02-18

**Authors:** Yu Zhang, Andrei A. Vakhtin, Jennifer S. Jennings, Payam Massaband, Max Wintermark, Patricia L. Craig, J. Wesson Ashford, J. David Clark, Ansgar J. Furst

**Affiliations:** 1 War Related Illness and Injury Study Center (WRIISC), VA Palo Alto Health Care System, Palo Alto, California, United States of America; 2 Psychiatry and Behavioral Sciences, Stanford University, Stanford, California, United States of America; 3 Radiology, VA Palo Alto Health Care System, Palo Alto, California, United States of America; 4 Neuroradiology at Stanford University, Stanford, California, United States of America; 5 Pain Clinic, VA Palo Alto Health Care System, Palo Alto, California, United States of America; 6 Anesthesiology, Perioperative and Pain Medicine, Stanford University, Stanford, California, United States of America; 7 Neurology and Neurological Sciences, Stanford University, Stanford, California, United States of America; University of Minnesota, UNITED STATES

## Abstract

Evaluation of brainstem pathways with diffusion tensor imaging (DTI) and tractography may provide insights into pathophysiologies associated with dysfunction of key brainstem circuits. However, identification of these tracts has been elusive, with relatively few in vivo human studies to date. In this paper we proposed an automated approach for reconstructing nine brainstem fiber trajectories of pathways that might be involved in pain modulation. We first performed native-space manual tractography of these fiber tracts in a small normative cohort of participants and confirmed the anatomical precision of the results using existing anatomical literature. Second, region-of-interest pairs were manually defined at each extracted fiber’s termini and nonlinearly warped to a standard anatomical brain template to create an atlas of the region-of-interest pairs. The resulting atlas was then transformed non-linearly into the native space of 17 veteran patients’ brains for automated brainstem tractography. Lastly, we assessed the relationships between the integrity levels of the obtained fiber bundles and pain severity levels. Fractional anisotropy (FA) measures derived using automated tractography reflected the respective tracts’ FA levels obtained via manual tractography. A significant inverse relationship between FA and pain levels was detected within the automatically derived dorsal and medial longitudinal fasciculi of the brainstem. This study demonstrates the feasibility of DTI in exploring brainstem circuitries involved in pain processing. In this context, the described automated approach is a viable alternative to the time-consuming manual tractography. The physiological and functional relevance of the measures derived from automated tractography is evidenced by their relationships with individual pain severities.

## Introduction

The brainstem, including the midbrain, pons, and medulla, involves structures with complex white matter pathways and gray matter nuclei that are concentrated in a small area. Intricate brainstem circuitries and nuclei serve systems such as respiratory and cardiovascular regulation, sleep and alertness, pain, posture, mood, and mnemonic functions. It is crucial to understand how structural changes in small brainstem regions and circuitries may cause/alter pathologies.

Studies of brainstem substructures using anatomical brain MRI have been complicated by difficulties in detecting neuronal loss due to a lack of sufficient contrast to delineate small internal substructures in intensity-based images. Diffusion tensor imaging (DTI) is a noninvasive MRI imaging technique that measures changes of extra-cellular molecular water diffusion in white matter microstructures [1,2]. Fractional anisotropy (FA), a commonly used DTI index, is known to be sensitive to detect damages in orientationally organized structures such as white matter tracts. Mean diffusivity (MD, s/mm^2^) is a scalar measure of the total diffusion with in a voxel that is not specific to gray or white matter tissue type. Axial (AD, s/mm^2^) and radial (RD, s/mm^2^) diffusivities each represent diffusion levels along or perpendicular to the principle axis, respectively, and can be used to detect pathological axonal or myelin states [3]. Furthermore, based on computing the directional information in each voxel of DTI, tractography can be used to reconstruct trajectories of white matter tracts that correspond to known neuroanatomy in 3-dimensional space [4,5]. Fiber density [6] of a trajectory, which is the number of probable streamlines per voxel, provides another quantification of white matter tracts. Diffusion tensor tractography has orientation-based contrasts and thus permits not only the anatomical illustration of neural pathways, but also quantifications of integrity and structural connectivity levels of specific pathways by estimating the microstructural or fiber indices along the reconstructed tracts.

Diffusion tensor tractography has been broadly applied in neurosurgical settings, such as navigating tumor resections to avoid damage to surrounding vital neural pathways [7] and providing guidance to targets of deep brain stimulation electrodes [8]. Quantitative DTI measures in tractography-derived fiber bundles have also been used for detecting microstructural deficits in multiple neurologic and psychiatric disorders, such as amyotrophic lateral sclerosis [9], cognitive impairments and Alzheimer’s Disease [10–12], and other neurologic and psychiatric disorders [13]. However, tracking and isolating brainstem pathways using DTI is challenging due to their spatial overlapping and crossing with other major tracts that project to cerebral cortices. Previous tractography investigations pertaining to the brainstem have focused on major fiber bundles between the brainstem and the cortex [14] that provided few details about specific fiber tracts within the brainstem. A voxel-wise tract-based spatial statistics (TBSS) [15] approach allows for comparisons of FA in “skeletons” within the brainstem. While this method can be fully automated, the TBSS approach analyzes data at selected high-FA voxel locations that do not necessarily belong to actual fiber tracts, and the measures at voxel level could additionally be inaccurate when FA is disrupted in the presence of disease. There are, however, increasing clinical needs for investigating brainstem fiber tracts and their mnemonic functional relevance. A recent study [16] has established a human brainstem tract atlas based on tractography from a large population of 488 young healthy subjects. This tractographic atlas may indeed benefit clinical neurosurgical interventions, but the neuropathological imaging applications of measuring FA in non-invasively isolated brainstem fiber tracts are yet unknown. As atlas-based analyses are sensitive to individual uncertainties of small fiber bundles, tractography based on native DTI data is still needed. Furthermore, the atlas was derived from data collected from healthy young adults using a high angular resolution diffusion imaging (HARDI) sequence [17], which is very difficult to implement in most clinical DTI scan protocols for neurological purposes. A reliable, clinically applicable, brainstem tractography and quantitative measure is thus needed.

The aims of this study were to 1) demonstrate the practical feasibility and neuroanatomical consistency of tractography for most trackable brainstem tracts based on a clinical DTI scan protocol; 2) automate the described tractographic procedure and test its reliability of quantitative measures of the brainstem tracts together with manual tractography; and 3) use automated tractography to assess the association between the integrity of brainstem tracts and chronic pain in an attempt to identify brainstem pathways that are specifically involved in pain regulation and processing.

## Materials and methods

### Participants and pain evaluation

Between 2011 and 2017, 326 veteran patients underwent the same MRI protocol at the California War Related Illness and Injury Study Center (WRIISC-CA) at the Veterans Affairs Palo Alto Health Care System (VAPAHCS). The study was approved by the Stanford University and VAPAHCS Institutional Review Boards, and written informed consent was obtained from each participant prior to study enrollment. After review of MRI images, the structural and diffusion scans obtained from 300 participants who presented no specific radiological abnormalities (e.g. tumor, stroke, multiple sclerosis plaques, etc.) or artifacts were entered into an in-lab computer post-processing pipeline, which corrected for DTI distortions, generated an MRI atlas in standard space, and transformed images between native and standard spaces.

From this imaging database, the current study, selected 17 participants (age range = 39–59 years; mean = 49.7 ± 5.2; 16 males) for brainstem tractography. Among these 17 participants, within 24 hours of undergoing MRI, 12 veterans filled out item No.6 from the Brief Pain Inventory Short Form (BPI) [18], which queried their current pain levels at that particular time (‘pain right now’); all 17 participants reported the worst pain they could recall in the month prior to MRI (‘worst pain in last month’). Both pain scores were recorded on a scale from 0 (none) to 10 (extreme) and were not specific to any particular location on the body.

### Structural MRI and DTI acquisition

Brain imaging data were collected at the Veterans Affairs Hospital in Palo Alto, CA, USA, using a 3 tesla GE Discovery MR750 scanner with an 8 channel GE head coil. High-resolution T1-weighted images (T1WI) were acquired using a 4:07 minutes three-dimensional spoiled-gradient recalled acquisitions (3D-SPGR) in steady state (136 sagittal slices, TR/TE = 7.3/3.0 ms; flip angle = 11°; field of view = 250 mm; slice thickness = 1.2 mm with 0.6 mm slice gap; acquisition matrix = 256 × 256; number of excitations = 1.0; resolution = 1.05 mm × 1.05 mm × 0.60 mm). T2-weighted images (T2WI) were acquired using a 2:21 minutes fast spin-echo (FSE) sequences with TR/TE = 7652/98.4 ms, with 0.45 x 0.45 mm^2^ in-plane resolution and 3.5 mm slice thickness, for 47 axial slices. The T2WI images were collected for correcting geometric distortions of DTI relative to the structural T1WI. Diffusion tensor imaging scans were acquired with a 2D single-shot EPI sequence with TR/TE = 6600/84.1 ms, 1 x 1 x 2.5 mm^3^ resolutions, with 59 contiguous axial slices to enable full brain coverage. Ten scans without diffusion gradients (b0) and scans along 60 sensitization directions using diffusion-weighting gradients (DWI) with b-value of 1000s/mm^2^ were acquired for DTI reconstruction. The scan time for the DTI sequence was 8:02 minutes.

### MRI processing pipeline

**Fig 1:**
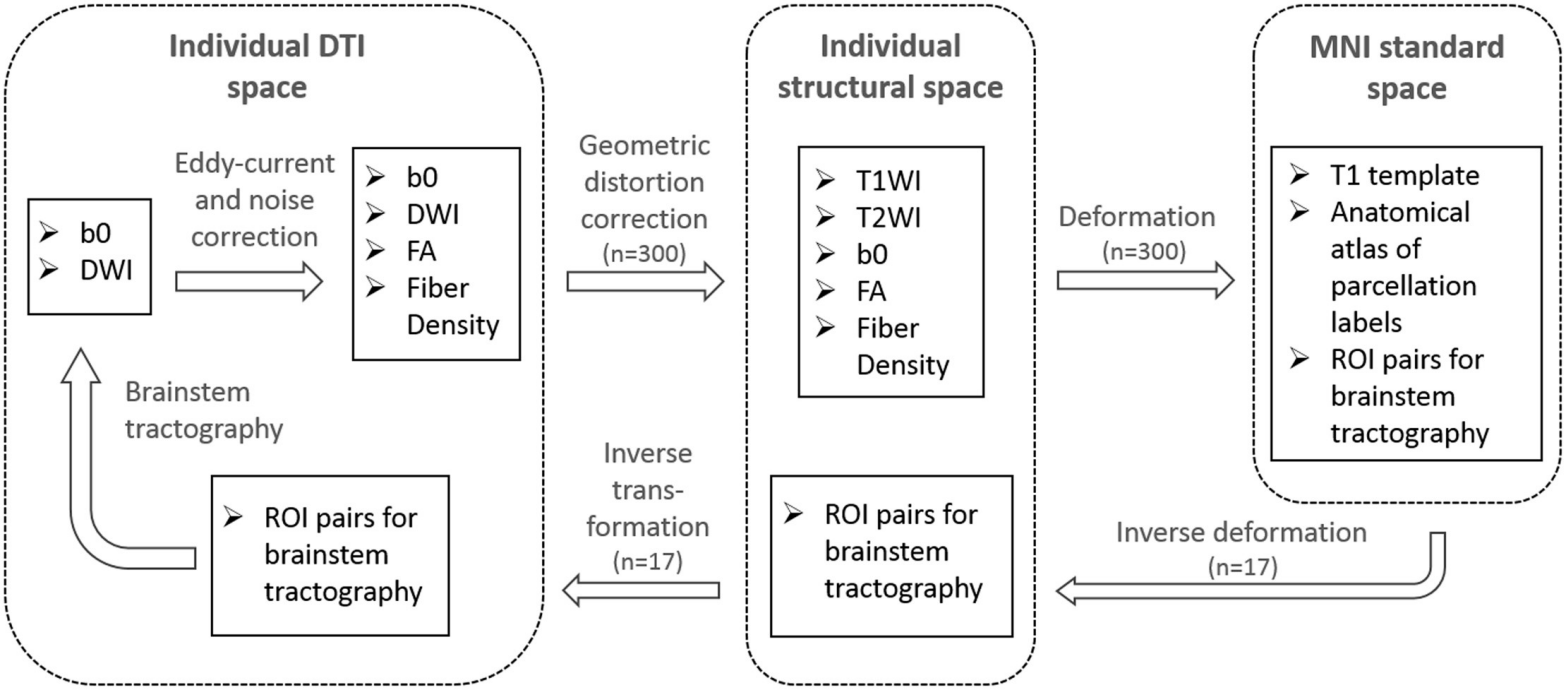
DTI processing and brainstem tractography flowchart. In-lab image processing pipeline for registration and atlas building based on structural MRI and DTI from 300 WRIISC-CA participants.

The T1WI and DTI scans were initially checked for visual artifacts, and subsequently processed using an in-lab image processing pipeline, shown in Fig 1. After quality control, structural (T1WI/T2WI) and DTI data sets collected from 300 WRIISC-CA participants, who were veterans aged between 30 and 80 years, were processed through this pipeline to create customized templates and atlases in Montreal Neurological Institute (MNI) space. Creating customized templates using averaged images from the same study cohort (e.g. WRIISC-CA) is recommended [19,20], because the minimal cross-subjects variation results largely improve the registration accuracy. This in-lab pipeline included the following: 1) correction for head motion, susceptibility artifacts, and eddy-current distortions in native space using scripts from the Stanford Vision Imaging Science and Technology Lab (VISTA Lab; https://vistalab.stanford.edu/software/). Maps of DTI metrics, such as fractional anisotropy (FA), mean diffusivity, radial diffusivity, axial diffusivity, were then computed using FDT software in FSL (https://fsl.fmrib.ox.ac.uk/fsl/fslwiki/FDT). 2) The eddy-current distortion correction in the first step was insufficient, because the geometric distortions of DTI relative to anatomical MRI (T1WI and T2WI) in subject’s native space, are still apparent in the brainstem. In this second step, we performed an additional distortion correction to correct the distorted EPI images onto non-distorted T1/T2WI. Briefly, we performed nonlinear registration of the first *b* = 0 *s/mm*^*2*^ image in each DWI set to its corresponding non-distorted T2WI structural image, and then to the non-distorted T1WI structural image, using a nonlinear deformation algorithm in Advanced Normalization Tools (ANTs) [21]. This procedure is similar to a second step distortion correction that described in a previous publication [22]. After the second step distortion correction, the non-linear distortions between DTI frame and anatomical T1WI were effectively reduced. Thus, the accuracy of anatomical landmark assignment within individual spaces was improved (S1 Fig). 3) Using nonlinear deformation algorithms in ANTs, all participants’ T1WI were diffeomorphically warped to the MNI space and averaged to create customized T1 and DTI templates. Then, an atlas with anatomical parcellations of whole-brain gray matter, white matter regions defined according to the JHU-DTI-MNI (Type I WMPM) [23] was spatially warped onto the customized template to provide anatomical labels for aiding the definition of regions-of-interest (ROIs) onto the templates. 4) The inverse of the above transformations was then used to transform anatomical parcellations and regions-of-interest (ROIs) specified in the MNI space to subjects’ native DTI space.

### Manual tractography of the brainstem tracts

**Fig 2:**
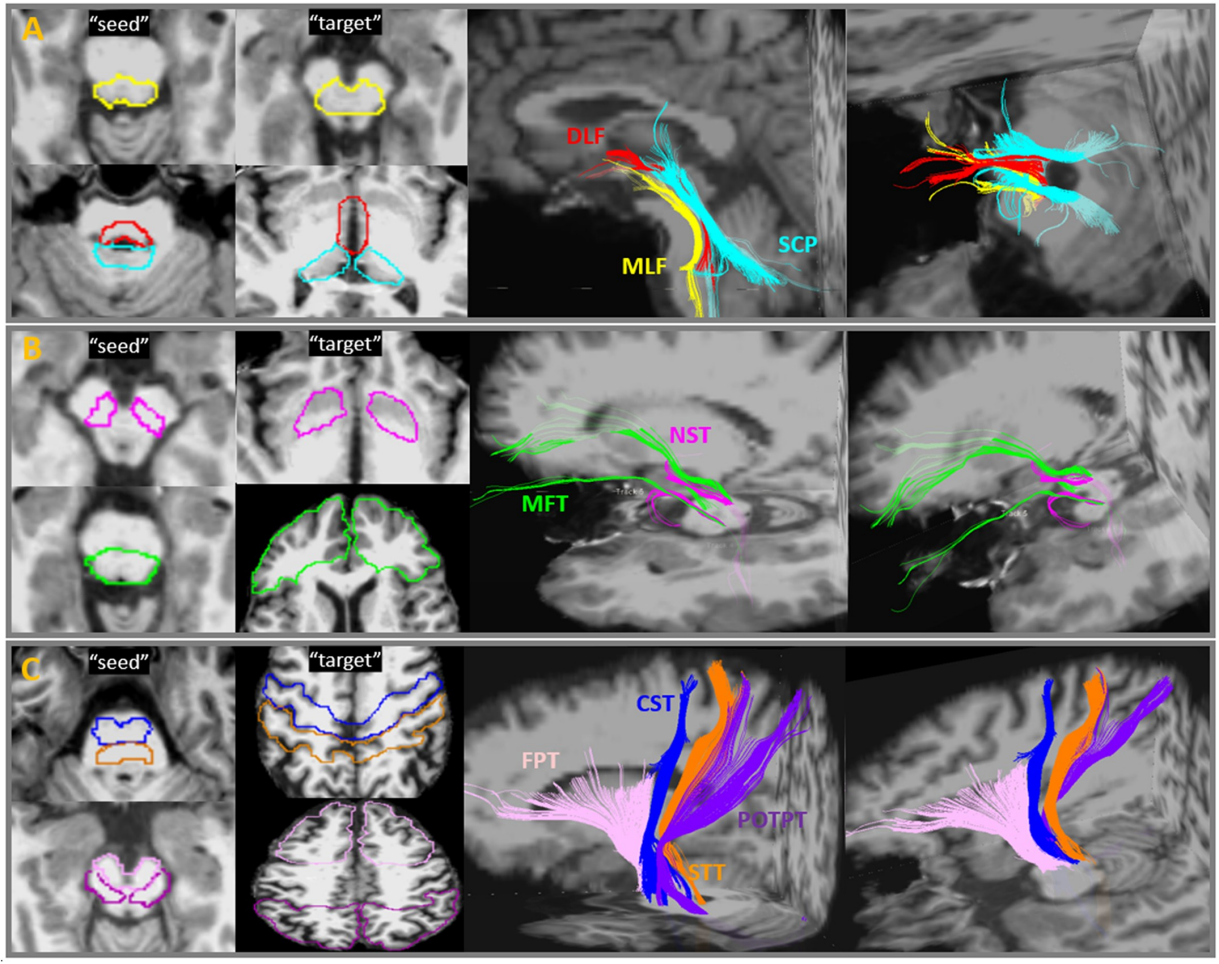
Brainstem fiber tracts and manual ROI placement. Placement of ROI-pairs (“Seed” and “target”) and their fiber outputs performed with manual tractography. Abbreviations: MLF = medial longitudinal fasciculus; DLF = dorsal longitudinal fasciculus; SCP = superior cerebellar peduncle; MFT = medial forebrain tract; NST = nigrostriatal tract; FPT = frontopontine tract; CST = corticospinal tract; STT = spinothalamic tract; POTPT = parieto-, occipito-, and temporopontine Tract.

**Fig 3:**
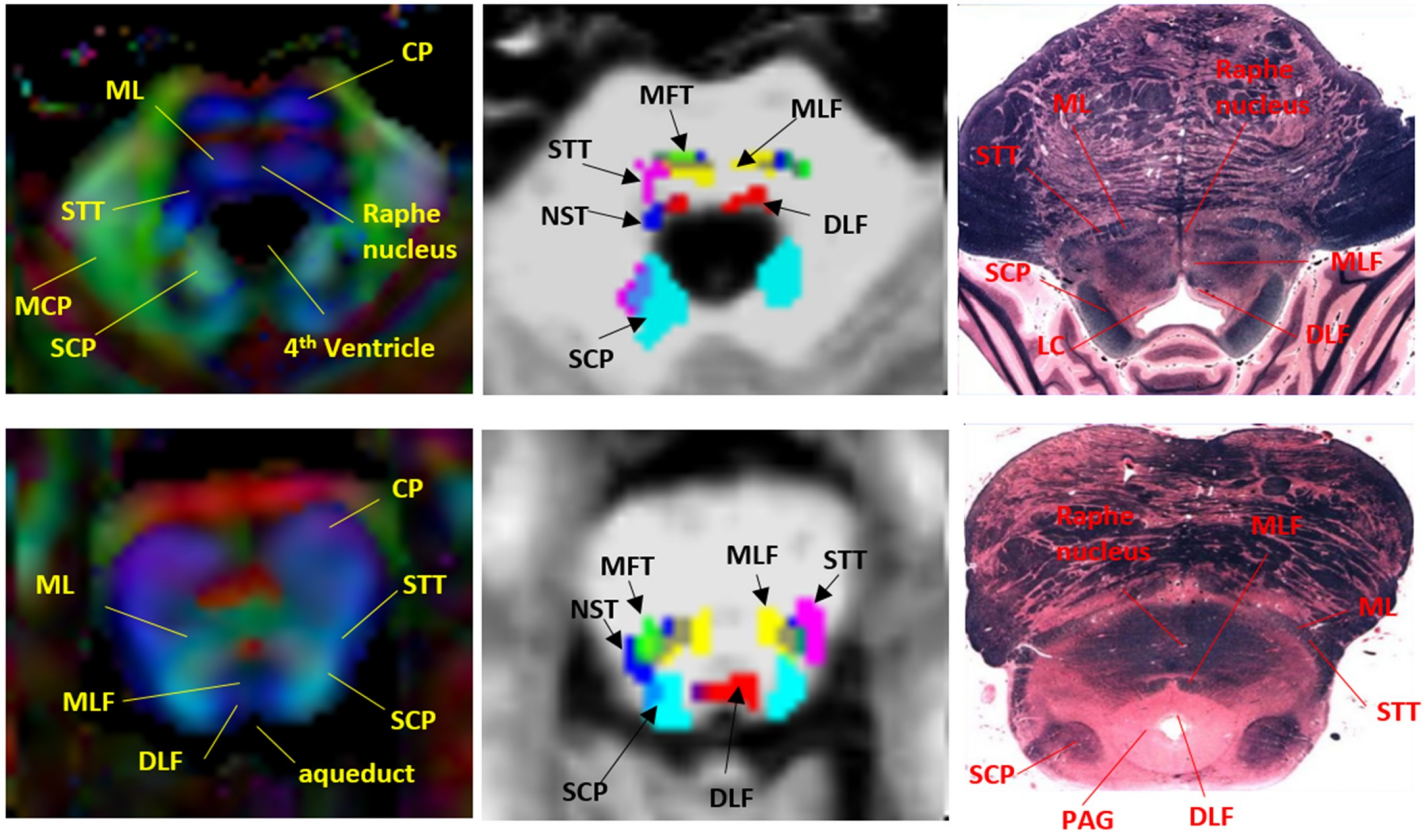
Representative brainstem fiber tracts and corresponding neuroanatomy in two brainstem levels. *Left*: color-coded anisotropic map (red: left-right oriented; blue: superior-inferior oriented; green: anterior-posterior oriented); *Middle*: six brainstem reticular tracts superimposed on a T1WI; *Right*: corresponding anatomic sections. Abbreviations: CP = cerebral peduncle; ML = medial lemniscus; STT = spinothalamic tract; MCP = middle cerebellar peduncle; SCP = superior cerebellar peduncle; MLF = medial longitudinal fasciculus); DLF = dorsal longitudinal fasciculus; MFT = Medial Forebrain Tract; NST = nigrostriatal tract; LC = locus coeruleus; PAG = periaqueductal gray matter. The brainstem anatomical pictures are downloaded from the web site (http://www.dartmouth.edu/~rswenson/Atlas/BrainStem/index.html) with permission of the author.

Manual brainstem tractography was performed on original b0 and DWI data using Diffusion Tensor Toolkits (DTK) (http://dti-tk.sourceforge.net/pmwiki/pmwiki.php) and TrackVis software (http://www.trackvis.org/), with a default linear least-squares fitting method. As a default function, DTK/TrackVis computed mean FA and fiber density (i.e. the streamline numbers) in each voxel of a fiber tract that resulted from tractography as the outcome measures. Each brainstem tract was initiated by placing a ‘seed’ ROI in the brainstem, and a ‘target’ ROI in the distant end. Deterministic tractography using a fixed step-length streamline propagation [24] algorithm was performed with an ‘AND’ option to reconstruct trajectories between both ROIs, with the minimum length and curvature thresholds of 10 mm and 30°, respectively. Seeds and targets were manually drawn by an experienced radiologist in accordance to known brainstem anatomy. The initial tractography consistently resulted in the intended trajectory, but additionally also resulted in other trajectories, some of which potentially being artifacts, and some that could be other neighboring pathways. In these cases, manual adjustments of ‘NOT’ ROIs were used to eliminate them. On the other hand, if the initial tractography had no trajectory results because the ‘seed’ and ‘target’ ROIs were too far apart, the distance between the ROI pairs was adjusted within their anatomical landmarks until any trajectories consistent with known anatomy could be achieved. Because manual tractography is extremely time-consuming, DTI data of 7 participants randomly selected from all participants were processed with manual tractography. Fig 2 depicts the ROI placements and reconstructed streamlines of nine fiber tracts that travel through the brainstem. For comparison with sectional anatomy, cross-sectional slices at the lower and upper brainstem panel of the color-oriented anisotropic map, six representative brainstem tracts, and corresponding neuroanatomy are shown in Fig 3. The nine pairs of brainstem fiber tracts were:

1. Medial longitudinal fasciculus (MLF; Fig 2A—yellow): the seed ROI was placed in the area anterior to the 4th ventricle floor, including the inferior cerebellar peduncle (ICP) and the medial lemniscus (ML). The target ROI was placed in the midbrain (MidB), encompassing the ventral tegmentum area (VTA) and the red nuclei (RedN). In the brainstem, the reconstructed fiber tracts travelled ventrally to the aqueduct and the 4th ventricle, slightly laterally to the midline, where the Raphe nuclei are located (Fig 3). This pattern was consistent with previous tractography of healthy human brainstems and known anatomy of MLF [16,24].
2. Dorsal Longitudinal Fasciculus (DLF; Fig 2A—red): the seed ROI was placed in the area anterior to the aqueduct, including the periaqueductal gray matter (PAG) and surrounding area as well as the locus coeruleus (LC). The target ROI was placed in the dorsal MidB, including the mammillothalamic tract, which is located laterally to the cerebral aqueduct. The reconstructed fiber tracts connected between the LC and the hypothalamus, travelled ventrolaterally to the cerebral aqueduct, and ran dorsocaudally to the MLF (Fig 3). The tractography pattern of DLF was consistent with known anatomy and a previously-described tractography atlas [16,25].
3. Superior Cerebellar Peduncle (SCP; Fig 2A—cyan): The seed ROI was placed in the area posterior to the 4th ventricle floor and aqueduct, including white matter in the superior cerebellar peduncle and the cerebellar dentate. The target ROI was placed in the ventral thalamus. The reconstructed fiber tracts connected the dentate nuclei and the thalamus, including non-decussated and decussated dentatorubrothalamic tracts. This tract and its anatomy have been reported in many previous tractography studies of the human brain [16, 26].
4. Nigrostriatal tract (NST; Fig 2B—pink): The seed ROI was placed in the MidB, including the entire substantia nigra (SN). The target ROI was placed in the inferior level of the putamen and the globus pallidus formation (Put/GP). This small fiber tract travelled from the SN dorsomedially to the subthalamic nuclei (STN) and turned dorsolaterally toward the ventral Put/GP. This pattern was consistent with previous tractography findings and known anatomy of the NST [27].
5. Medial Forebrain Tract (MFT; Fig 2B—green): The seed ROI was placed in the ventral MidB area anterior to the aqueduct, including ventral tegmental area (VTA). The target ROI was placed in the inferior frontal gray and white matter area, including the rectus, orbitofrontal, and the prefrontal cortices. This tract originated in the VTA, extended dorsolaterally toward the nucleus accumbens (NAc) and anterior thalamic radiation (ATR, located in the anterior limb of the internal capsule), and finally projected to the orbitofrontal cortex (OFC) and the dorsolateral prefrontal cortex (DLPFC). The tractography and neuroanatomy of MFT has been demonstrated in previous papers [28,29].
6. Frontopontine Tract (FPT; Fig 2C—light pink): The seed ROI was placed in the ventral medial 1/3 of the cerebral peduncle (CP). The target ROI was placed in the middle and superior frontal gray and white matter, including the supplementary motor and the premotor areas, but excluding the primary motor area. The FPTs, originate from the frontal lobe, descend through the anterior limb of the internal capsule, and end in the pontine nuclei, have been described in many neuroanatomy textbooks and tractography studies [30].
7. Corticospinal Tract (CST; Fig 2C—blue): The seed ROI was placed in the middle 1/3 of the CP. The target’ ROI was placed in the precentral gray and white matter, which corresponds to the primary motor area. The CST is the primary motor pathway descending from the motor cortex innervating muscles controlling skilled voluntary movement. This ROI placement of tracking the CST is in line with previously well-described strategies to reconstruct CST using DTI tractography [30].
8. Spinothalamic Tract (STT; Fig 2C—orange): The seed ROI was placed in the ML and the surrounding area. The target ROI was placed in postcentral (sensory) gray and white matter, corresponding to the primary somatosensory area. The STT rose up the brainstem ML area, projected to thalamus, and eventually reached the sensory cortex. The tractography of this pathway has been demonstrated previously [16].
9. Parieto-, Occipito-, and Temporopontine Tract (POTPT; Fig 2C—purple): The seed ROI was placed in the laterocaudal third of the CP. The target ROI was placed broadly in the parietal, occipital, and temporal gray and white matter parcellations. The parietopontine and occipitopontine tracts passed the posterior limb of the internal capsule, retrolenticular part of the internal capsule (RLIC), and the dorsal thalamus; the temporopontine tract travelled medially to the hippocampoamygdala formation. These patterns were in agreement with a previous report [16].

The tractographic anatomy, possibly related neurotransmitter systems and functions of the nine tracts reconstructed from diffusion tensor tractography are summarized in Table 1.

**Table 1:** Anatomy along with possible associated neurotransmitters and functions, as well as associated references of brainstem circuits.

Brainstem tracts	Gray/white matter structures in the brainstem tractographic pathways	Possible Neuro-transmitter	Related Functions	Associated References
MLF	Begin: ICP and ML;	Serotonin, Noradrenaline	wakefulness, pain control, maintaining posture, cardiovascular control, eye movement	[31–33]
	Pass: lower raphe nuclei, ML;			
	End: MidB and RN			
DLF	Begin: PAG and Surrounding area;	Serotonin, Noradrenaline,	wakefulness, attention, consciousness, stress and reward, pain	[34–40]
	Pass: LC, PAG;			
	End: hypothalamus; mamillary body			
SCP	Begin: SCP and cerebellar dentate nuclei;	Acetylcholine, GABA	sleep, cognition, mood, attention, arousal, voluntary limb movements, locomotion	[41–44]
	Pass: PPN;			
	End: RN and thalamus			
NST	Begin: SN	Dopamine, Serotonin	motor function, mood, pleasure and reinforcement	[40,45–47]
	Pass: VTA, STN			
	End: putamen and GP			
MFT	Begin: MidB and VTA	Acetylcholine, Dopamine	mood, word, reward and pleasure	[48–51]
	Pass: NAc, ATR			
	End: rectal, orbitofrontal, and prefrontal cortices			
FPT	Begin: medial 1/3 of the CP	Non-reticular system	control of movement, facial movement	[52,53]
	Pass: ALIC, SCR, frontal cortices			
	End: superior frontal, supplementary motor area, and the premotor areas			
CST	Begin: middle 1/3 of the CP	Non-reticular system	movement of body muscles	[54]
	Pass: PLIC			
	End: precentral (primary motor) cortex			
STT	Begin: ML	Non-reticular system	sensory of temperature, pain	[55,56]
	Pass: dorsal spinal cord, ML, thalamus			
	End: postcentral (sensory) cortex			
POTPT	Begin: lateral 1/3 of the CP	Non-reticular system	variety of functions: working memory, learning, visual, auditory	[57,58]
	Pass: ventral medulla, RLIC, dorsal thalamus, amygdala and hippocampus			
	End: parietal, occipital, and temporal cortex			

Abbreviations: ICP = inferior cerebellar peduncle; ML = medial lemniscus; MidB = midbrain; RN = red nucleus; PAG = periaquaductal grey matter; LC = locus coeruleus; SCP = superior cerebellar peduncle; PPN = pedunculopontine nucleus; SN = substantia nigra; VTA = ventral tegmental area; STN = subthalamic nucleus; GP = Globus pallidus; NAc = Nucleus Accumbens; ATR = anterior thalamic radiation; CP = cerebral peduncle; ALIC = anterior limb and genu of the internal capsule; SCR = superior coronal radiata; PLIC = posterior limb of the internal capsule; RLIC = retrolenticular part of the internal capsule.

### Automated brainstem tractography

A common practice of automated-tractography is to apply pre-defined ROI-pairs from the MNI atlas space onto individual DTI data to extract only those streamlines that run through both ROI ends. However, most of the brainstem fiber tracts (e.g. MLF, DLF, NST and MFT) do not have a previously established ROI atlas or parcellations in MNI space. In this study, we use the manually-defined ROI-pairs and tractographic results to build the pre-defined ROI templates of the brainstem tracts and subsequently tested the reproducibility of these tractography results in comparison with manual tractography. Briefly, the procedure of building the templates of ROI-pairs in MNI space included: 1) employing the in-lab imaging processing pipeline to transfer the manually defined ROI pairs and the 9-brainstem tracts into MNI space. 2) in MNI space, the normalized ROI pairs of each tract were averaged and binarized to create the pre-defined ROI-pair templates; the normalized tracts were also averaged to create tract templates as the anatomical references; 3) to further improve anatomical precision of each tract isolation, minimal adjustments of the ROI templates were performed including: (i) dilating the ROI boundaries to ensure the ROI-pairs fully cover the termini of each fiber tract’s template, (ii) eroding ROI boundaries between two adjacent ROIs (e.g. the boundaries of MLF and DLF) to eliminate overlapping tractographic results; (iii) limiting the dilation and erosion of the ROI templates within the anatomical landmarks based on the JHU-DTI-MNI atlas (also described in Table 1 as the ‘begin’ and ‘end’ regions). Once the templates of ROI-pairs were built in MNI space (see S1 Table for x-y-z coordinates and voxel sizes of ROI pairs), they were transferred backwards through the inverse transformation of the in-lab image processing pipeline onto all participants’ native DTI space to launch individual brainstem trajectories between the assigned ROI-pairs. The brainstem tractographic outputs after using automatically assigned ROI-pairs did not show systematic mislabeling of the adjacent tracts in any of the 17 participants, eliminating the need for additional manual adjustments using “NOT” ROIs at the time of this study. The overall processing time of this automated tractography procedure for tracking the 9 brainstem tract pairs was approximately 30~40 minutes per subject.

### Outcome measures

There is currently no comparative standard for determining reliability of the tractographic results. Regardless of tractographic approaches, the no generation (absence) of expected fiber lines is still a problem (i.e. false negative tractography) that can result in failure of quantitative outcome measurements along existing tracts. The ability to display an existing fiber tract consistently across a group of subjects is considered a good indicator of the reliability of a given technique: A recent review study [59] used this metric, called ‘success rate’, to express a ratio of the number of generated tracts over the number of expected tracts based on the existing literature. For example, if we know from the literature that a given brain region contains a specific fiber tract and we find this tract in 80 out of 100 subjects then the success rate is 80%. Conversely, if the tract in question was not identified in any of the subjects the success rate would be 0%, indicating a complete failure of the technique. Similarly, in this study, we calculated the ‘success rate’ as a percentage of the number of subjects where fiber tracts were identified successfully (n ×1/2 for unilateral tract identification, n ×1 for bilateral tract identification) divided by the total number of subjects on which a given technique was performed. This resulted in success rates for manual and automated approaches separately.

To date, manual tractography is still an important tractographic approach, because manual ROI placements by neuroanatomical experts provide high anatomical precision. Furthermore, the unexpected fiber lines (i.e. false positive tractography), if there are any, can be manually removed based on prior anatomical knowledge. Although manual tractography is imperfect, it is a conventionally accepted validation method [59]. Therefore, if the automated tractography reliably reproduce the manual tractography, then the automated approach could be considered an alternative approach to the manual tractography. In each presented brainstem tract, two quantitative scalar indices were obtained as the major outcome measures: (1) The FA index, which indicates the microstructural integrity of fiber connection, based on the mean FA per 1 mm^3^ voxel in each brainstem tract. The fiber density index, which represents the weights of reconstructed fiber tracts, based on the mean streamline numbers per 1 mm^3^ voxel in each brainstem tract [60]. We used an intra-class correlation coefficient (ICC) to test if quantitative measures based on automated tractography could reliably reproduce the same measure based on manual tractography.

In addition to FA index, diffusivity indices including MD, RD and AD were also measured in each presented brainstem tract for examining the relationship between DTI and pain levels.

### Statistical analyses

All statistics were performed in SPSS (IBM SPSS Statistics for Windows, Version 24.0. Armonk, NY: IBM Corp.). For n = 7 participants who underwent both manual and automated tractography, an intra-class correlation coefficient (ICC) model in the reliability analysis was used to test reliability between automated and manual tractography, for each brainstem tract, as measured by FA and fiber density, respectively. For the 17 participants who had quantitative outcome measures of the brainstem tracts, linear regression models were initially used to examine the relationships of FA and fiber density in each brainstem tract with the two self-reported pain levels (i.e. ‘pain right now’ and ‘worst pain last month’), separately. In the linear regression models, DTI quantitative measurements were the dependent variables, pain levels were the predictors, and age and sex were the covariates. The results of the linear relationships were reported as B (the coefficient of changes per pain scale increasing), R-squared, Cohen’s *d* effect sizes [61], and 95% confidence interval for B. Because sample size was small, bootstrapping with n = 1000 was reperformed on this linear regression model for testing the relationships between pain levels and all DTI indices including FA, MD, RD and AD. A small number of failed tractography and unavailable pain scores were included in all statistic models as missing values. For all above tests, the significant level was *p* ≤ 0.05. In addition, a false recovery rate multiple comparison resulted a critical significant level for adjusted-*p* ≤ 0.026.

## Results

### Success rate

S2 Table lists the success rates of manual and automated tractographic performances in each brainstem tract. For manual tractography, although most of the brainstem tracts were successfully presented, a few failed tractography occurred in fibers that travelled through long distances or areas susceptible to artifacts, crossing-fibers and other quality problems. Automated tractography achieved a slightly smaller, but similar success rate as the manual tractography. Both approaches had 85–100% success rates, which suggest that brainstem tractography based on our conventional DTI quality may be feasible.

### Reproducibility of quantitative tractography

S3 Table also summarizes reproducibility between manual and automated tractographic approaches. The FA in tracts measured by automated tractography reliably reproduced those measured by manual tractography. On the other hand, fiber density from automated tractography could marginally reproduce the results from manual tractography.

### Relations between DTI and pain levels

**Fig 4:**
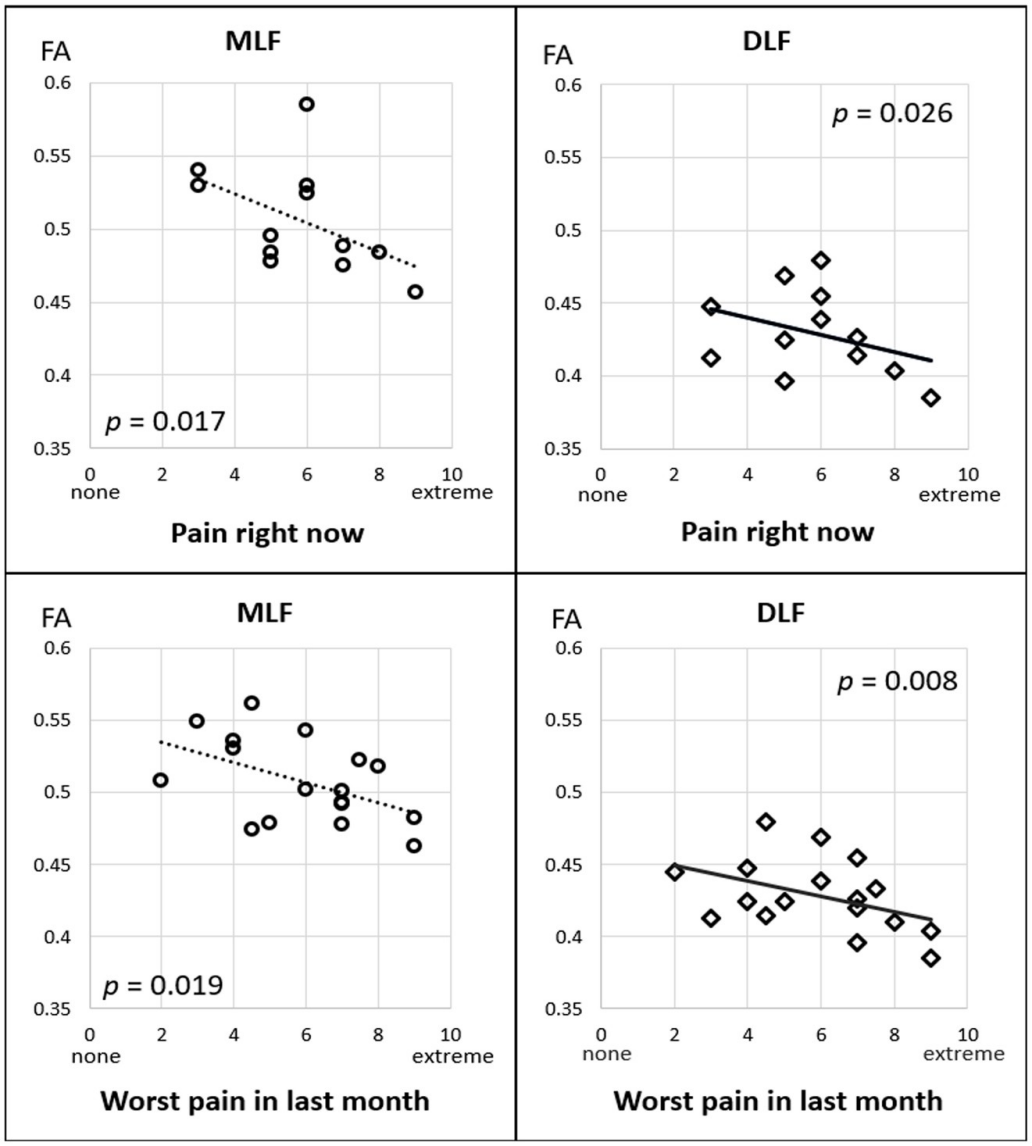
Relations between FA and pain levels. Scatter plots of relations between FA of the MLF and DLF tracts and two pain scales.

Linear regression models were used to examine the relationships of FA or fiber density in each brainstem tract with the two self-reported pain levels (i.e. ‘pain right now’ and ‘worst pain last month’), separately. Table 2 and Fig 4 show that, significant associations were observed between both pain levels and FA in the DLF and MLF, but not in other brainstem tracts. Specifically, after controlling for age and gender, per unit increase in ‘pain right now’ level corresponded to a 3.48% (CI_95_: 0.5 to 6.4%, *p* = 0.026) FA reduction in DLF and a 3.02% (CI_95_: 0.7 to 5.4%, *p* = 0.017) FA reduction in MLF. Furthermore, per unit increase in ‘worst pain last month’ corresponded to a 2.30% (CI_95_: 0.7 to 3.9%, *p* = 0.008) FA reduction in DLF and 1.87% (CI_95_: 0.4 to 3.4%, *p* = 0.019) FA reduction in MLF. No significant relation between pain levels and fiber density was detected.

**Table 2:** Linear regressions between FA of brainstem tracts and pain levels.

Tract name		Pain Right Now (n = 12)				Worst Pain Last Month (n = 17)		
	Mean (S.E.) % FA change rate /scale increase	R-Squared	Effect-size	*p*-value	Mean (S.E.) % FA change rate /scale increase	R-Squared	Effect-size	*p*-value
**MLF**	**-3.02 (1.04)**	**0.533**	**-1.75**	**0.017**	**-1.87 (0.71)**	**0.346**	**-1.32**	**0.019**
**DLF**	**-3.48 (1.30)**	**0.664**	**-1.61**	**0.026**	**-2.30 (0.75)**	**0.487**	**-1.53**	**0.008**
**SCP**	-7.32 (3.28)	0.655	-1.34	0.053	-2.98 (1.47)	0.318	-1.02	0.062
**NST**	-0.72 (1.50)	0.503	-0.15	0.644	-2.47 (1.83)	0.394	-0.75	0.204
**MFT**	0.94 (0.62)	0.349	1.07	0.180	0.30 (0.53)	0.293	0.33	0.582
**FPT**	-0.60 (0.82)	0.095	-0.47	0.482	0.35 (0.63)	0.076	0.29	0.588
**CST**	0.21 (1.05)	0.046	0.12	0.860	0.84 (0.95)	0.242	0.51	0.393
**STT**	-0.59 (0.66)	0.388	-0.53	0.400	0.01 (0.68)	0.228	0.01	0.989
**POTPT**	-0.79 (1.39)	0.105	-0.38	0.586	-0.04 (1.12)	0.226	-0.02	0.970

Mean (S.E.) FA change rate (%) associated with per increased pain scale, effect size and significance that estimated based on linear regression test for each brainstem fiber tract.

Effect size = (2 * T-value) / SQRT (degree of freedom)

**Bold**: linear regression was significant with a multiple comparison-adjusted critical *p* threshold.

S4 Table shows associations between the four DTI microstructural indices and pain levels based on bootstrapped linear regression tests. After bootstrapping, a weaker, but still significant, negative association remained between FA and pain in the DLF and MLF. Non-FA diffusivity indices of the brainstem tracts did not show robust pain-related changes, except for a significant relationship between decreased AD and increased pain levels.

## Discussion

In this study, we introduced an automated tractographic approach based on a predefined ROI atlas for efficiently tracking and isolating small white matter tracts in the human brainstem. Our results demonstrated the usefulness of quantitative tractography in analyzing the relationship between brainstem tracts and chronic pain. This application was used to detect several brainstem tracts and diminished white matter integrity levels were associated with pain severity.

### Brainstem tractographic anatomy and neurological implications

**Fig 5:**
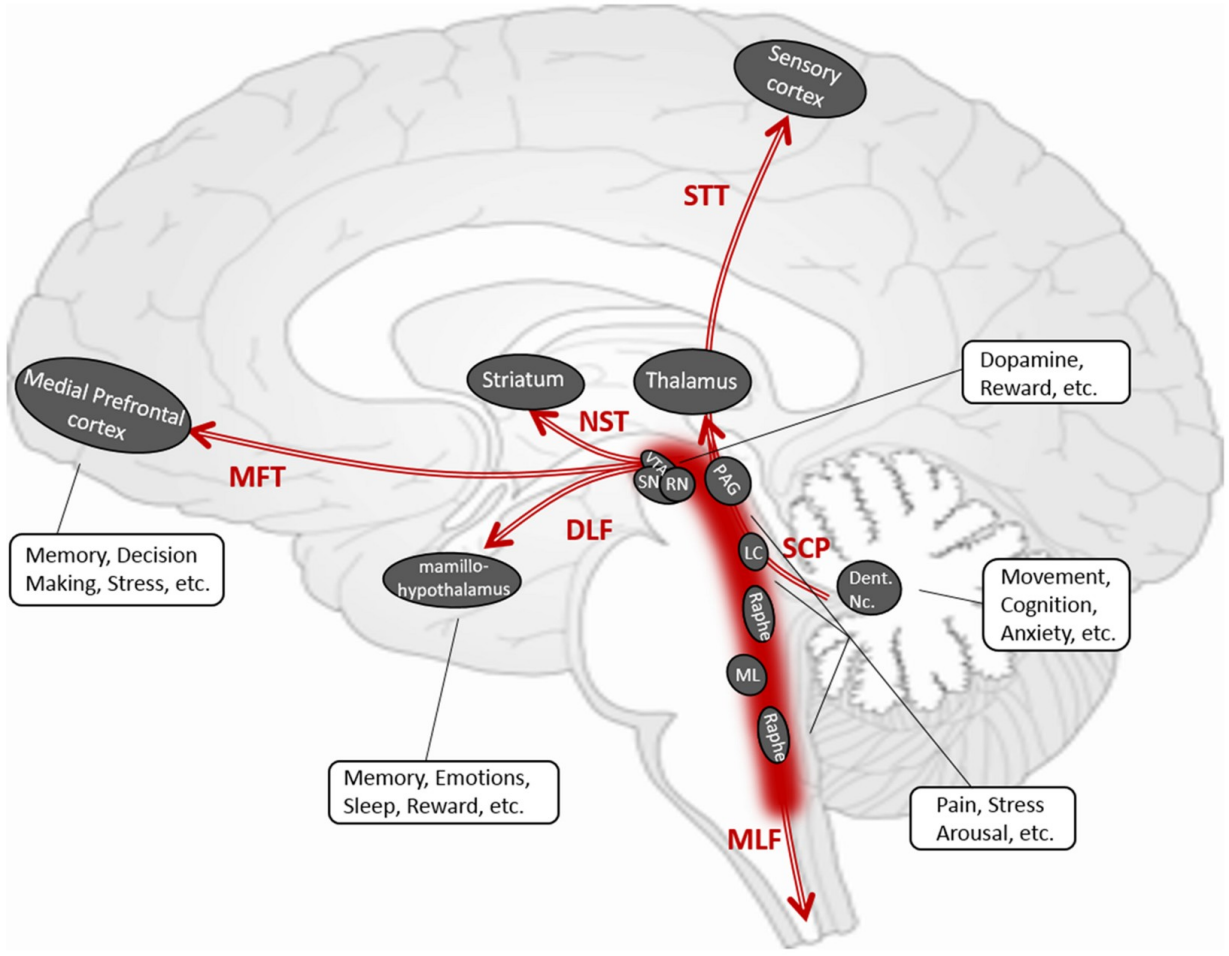
Illustration of brainstem reticular tracts. Conceptual sketch according to the DTI tractography of the brainstem reticular tracts, their anatomy, fiber-linked gray matter centers and potentially associated functions.

Most brainstem fibers could be subdivided into three types of circuits: 1) the corticopontine fibers that travel between spinal cord, pons, and the neocortices, including FPT, CST, STT, POTPT (Table 1 and Fig 2C); 2) the reticular tracts that interconnect nuclei that are located throughout the brainstem and the midbrain, including DLF, MLF, SCP, MFT and NST (Table 1 and Fig 2A and 2B); and 3) the pontinopeduncle tracts that run transversely between pons and cerebellar peduncles including the middle cerebellar peduncle, the inferior cerebellar peduncle and pontine crossing tracts. Among this traffic, the reticular tracts have major clinical significance in indicating essential neurotransmitter functions (Table 1 and Fig 5). Manual and automatic tractography of the corticopontine fibers and pontinopeduncle tracts have been well-established previously. By contrast, tractography of the reticular tracts needs to be further established as these fibers are thought to contain a variety of autonomic functions through corresponding neurotransmitter pathways.

MLF is generally known to connect the cranial nerves (III, IV, and VI) which are involved in eye movement, as well as the stabilization of the head and neck. MLF is also known to send parasagittal fibers from the dorsal raphe nuclei, which serve as a center of serotonergic neurons [33]. The role of MLF in modulating some autonomic, reproductive and excretory functions, as well as modulating perception to pain needs to be investigated. The DLF that links the hypothalamus, periaqueductal gray matter, locus coeruleus, and the nucleus solitarius in the medulla, carries both ascending and descending fibers. This tract is considered to be a pathway regulating the sleep–wake cycle, mood, and cognition [35,39]. DLF is also related to pain control [62]. SCP is a large bundle of projection fibers arising chiefly from the cerebellar dentate nuclei, ascending along the posterior floor of the forth ventricle, and ending in the red nucleus and thalamus. SCP plays a major role in the coordination of movement of the ipsilateral limbs. Previous studies [63,64] also suggest that SCP contains GABAergic fibers. The NST is a dopaminergic pathway that is involved in Parkinson’s disease. Individual tractography-guided quantitative DTI measures of this tract have been suggested as a promising marker for identifying Parkinson’s disease and such measurements may correlate with the severity of motor dysfunction [65–67]. NST is also suggested to control depressive fatigue states in Parkinson’s Disease [68,69]. MFT, which connects the brainstem to nucleus accumbens, anterior thalamic radiation, and dorsal and medial prefrontal cortices, is involved in mood, the reward system, the appetitive motivational seeking system and euphoria [70]. The relation of MFT and major depressive disorder has been well-documented through DTI [28], and MFT tractography is also used for targeting deep brain stimulation in depression [29].

Given the variety of the reticular fibers and their corresponding functions, brainstem DTI tractography may be highly informative in the diagnosis and study of a range of neurological conditions. Our newly developed brainstem tractographic approach allows the correlation analyses for clinical measures and quantitative DTI measures of fiber tracts known to be involved in those neurological dysfunctions.

### Quantitative brainstem tractography and pain

Much of the DTI and FA studies have been focusing on examining differences in white matter microstructure in chronic pain vs. non-pain controls, and correlating FA with pain levels (see a recent review paper [71]). Based on TBSS, pain-related FA reductions have been detected in multiple white matter tracts along diffuse ‘white matter skeletons’, including the internal and external/extreme capsules, corpus callosum, cingulum, thalamic radiation, and brainstem white matter, primary somatosensory and motor cortices, orbitofrontal cortex [72,73], rostral anterior cingulate, and dorsal lateral prefrontal cortex [74]. TBSS and most voxel-based analytic approaches, however, are known to be highly sensitive to registration errors and may produce false positive findings [75]. Moreover, alterations in most cerebral white matter regions could also be observed in comorbid neurological symptoms such as cognitive or mood impairments, but are not specifically responsible for pain [76]. In this context, it is essential to identify key white matter circuits involved in pain perception and regulation, and to explore the potential disruptions of these key pathways in relation with pain severity levels. Electrophysiological [77] and functional MRI studies [78] have shown that stimulus-evoked pain is associated with activity in several brain areas (‘pain centers’) and pathways linking these areas. However, these observations did not address the actual tracts and their structural connections in association with pain states. According to brainstem anatomy, both the ‘ascending (bottom-up) pathway’–which sends the nociceptive flow from the spinal cord to cerebral cortices, and the ‘descending (top-down) pathway’–that supports a top-down role in modulating the nociceptive signals, are involved in the brainstem fiber traffic [79]. A previous DTI study [80] demonstrated that fiber tracts originating in the periaquaductal grey (PAG) that project towards other brain areas, which may represent the descending pain control pathway, can be successfully visualized using probabilistic tractography. However, this study did not provide specific fiber isolation and quantitative measures such as FA in subjects with pain. Our study further isolated this theoretical ‘descending pathway’ to mainly overlap with the DLF and MLF tracts. We found that diminished FA in these tracts is associated with both ongoing pain (i.e. ‘pain right now’) and chronic pain (i.e. ‘pain in last month’) levels. These findings suggest that quantitative brainstem tractography is a promising marker for investigating pain regulation mechanisms. In addition, we also observed a strong association between reduced AD and increased pain levels in the MLF. This finding could be complementary to the FA data because: (1) AD change is believed, at least in part, to correlate with FA change because FA is a scalar that is partially derived from AD; (2) animal studies suggest that AD decreases correlates with axonal injury in spinal cord [81]. Further analyses based on larger patient populations and healthy controls are needed to validate these preliminary findings.

#### Advantages of automated brainstem tractography

This newly developed automated brainstem tractography has several merits: First, this approach has been shown feasible to be applied, implemented and performed on conventional DTI scans. Tracking the brainstem functional fibers on DTI is known to be difficult, and the detection of those small fiber anatomies depends on to the quality of acquisition including resolution, directions, controlling of noise and crossing-fibers. Existing approaches that provide comprehensive brainstem tractography have been established based on sophisticated diffusion acquisitions such as HARDI, diffusion spectrum (DSI) [82], etc., which provide limited clinical utility. In this study, we applied brainstem tractography using a readily available and sufficiently short (8 min) conventional DTI sequence, and demonstrated efficient success rates based on either manual or automated tractographic approaches. This suggest that the automated tractography could be adopted for quantitative measures in most clinical DTI protocols that use conventional resolutions (4~8 mm^3^ voxel size) and directions (30~80). Second, this automated tractographic approach provides reproducible measures that overcome known difficulties associated with manual tractography. Manual brainstem tractography is time consuming, depends on individual experience, and may suffer from reproducibility problems. Most existing automated tractography programs [14,83–85] have been used for representing tract trajectories of several major cerebral pathways with known anatomy. Automated brainstem tractography is currently not available due to a lack of atlas-segmented ROIs of brainstem small regions. Our study developed this automated tractographic approach based on pre-defining ROI pairs in the standard MNI space, with a combination of a high-quality DTI and MRI image registration pipeline. We demonstrated that this approach achieves similar FA measures as manual tractography while offering significant benefits through elimination of subjective rater bias, greatly reduced processing times, and the feasibility for quantitative analyses of large numbers of DTI data.

### Limitations

The work presented in this paper has several limitations: first, we did not identify all brainstem tracts: 1) Tractographic visibility is subject to the quality of DTI scans. Small tracts, such as the rubrospinal and the central tegmental tracts, previously addressed by Meola and colleagues [16], are not identifiable due to the limited quality of the DTI scan protocol used herein. 2) Other pain-specific fibers connecting the PAG to cerebral cortices, described by Hadjipavlou and colleagues [80], were not included in our approach, because the anatomical certainty and practical feasibility of these fibers still need to be validated. 3) We did not include the pontinocerebellar, pontinomedulla, pontinospinal tracts, or cranial nerves into this approach due to the coverage of the brain scans and consideration of the susceptibility artifacts near the skull-base that may confound local fiber tracking. 4) We also did not perform tractography of the middle or the inferior cerebellar peduncles, as well as the pontine crossing tracts because of a lack of knowledge regarding the physiological and functional relevance of these transverse fibers. A second limitation is the low image resolution. Most of the clinical MRI scan protocols have been limited in scan time and could not provide isotropic, high resolution DTI scans or additional field maps. Thus, the pre-defined ROI atlas maybe only feasible for clinical DTI data with low resolution, whereas ROI sizes and contours have to be readjusted if applied onto high-angular, high-resolution diffusion sequences, or other tractographic approaches (e.g. probabilistic tractography or Orientation Distribution Function (ODF)-based tractography. Another limitation of this study is the small sample size. We did not present the multiple comparisons-corrected significance this study is a preliminary, exploratory design and adequate “a priori" sample size calculations were not feasible. Further validation is warranted using this automated tractographic approach in larger populations, including more detailed pain evaluations and a more comprehensive consideration of all available DTI metrics. Nevertheless, quantitative DTI changes of specific brainstem fibers provide a promising biomarker in chronic pain.

### Conclusions

This study developed an automated approach for tracking brainstem fibers based on DTI. This approach is feasible for quantitative analyses and is an alternative to the very time consuming manual tractography. We demonstrated the feasibility of this approach in correlating the disruptions of some key brainstem tracts with individual pain severities. Further applications of this brainstem tractographic approach may aid in the exploration of clinical implications of these fibers in association with chronic pain syndromes, sleep disturbances, mood alterations, autonomic disturbances and other neurological disorders.

## Supporting information

S1 Fig Example of distortion corrections through image processing pipeline. Intrinsic distortion correction between individual FA map (green color) and structural T1WI (gray color) improves registration accuracy in brainstem area (arrowed and zoomed areas).
(DOCX)

S1 Table The pre-defined ROI atlas in MNI space. MNI coordinates and number of voxels for each pre-defined ROI pair.
(DOCX)

S2 Table Success rates based on manual and automated tractographic approaches. The success rates of each manual and automated tractographic performance.
(DOCX)

S3 Table Results of test-retest for the brainstem tractographic approaches. The reproducibility between manual and automated tractography based on the FA and fiber density measurements, respectively.
(DOCX)

S4 Table Linear regressions between DTI indices of brainstem tracts and pain levels. Estimated mean (S.E.) FA, mean, radial and axial diffusivity (MD, RD and AD) change rate (%) associated with per increased pain scale, R- Squared, effect size and significance that estimated based on bootstrapped-linear regression test for each brainstem tract.
(DOCX)
