# Supplementary material for: Diffusion tensor tractography of brainstem fibers and its application in pain
Source: PLoS One. 2020 Feb 18;15(2):e0213952. doi: 10.1371/journal.pone.0213952 (PMC7028272; doi:10.1371/journal.pone.0213952)

**Supplementary Figure S1**. Intrinsic distortion correction between individual FA map (green color) and structural T1WI (gray color) improves registration accuracy in brainstem area (arrowed and zoomed areas).


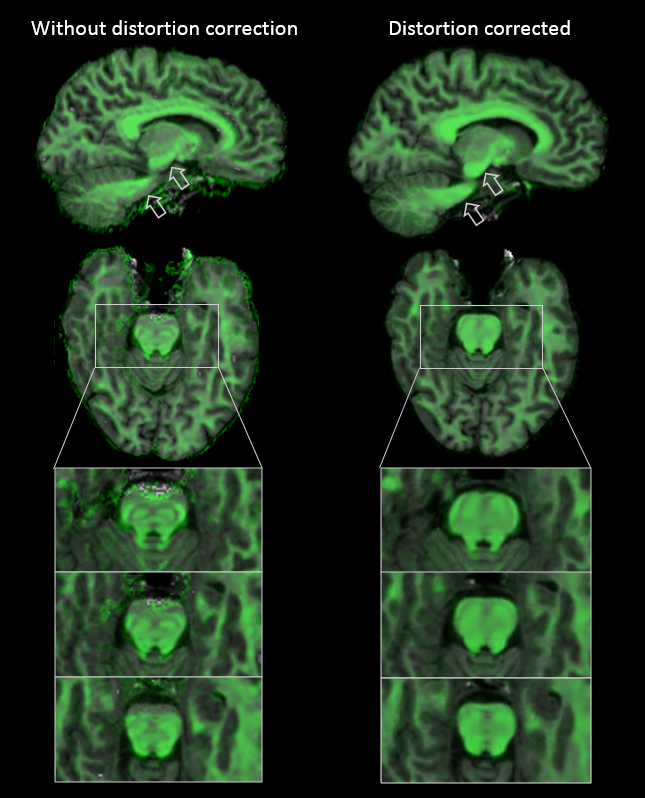

Supplement: S1 Fig — Intrinsic distortion correction between individual FA map (green color) and structural T1WI (gray color) improves registration accuracy in brainstem area (arrowed and zoomed areas). (DOCX) [file pone.0213952.s001.docx]
