# Supplementary material for: Diffusion tensor tractography of brainstem fibers and its application in pain
Source: PLoS One. 2020 Feb 18;15(2):e0213952. doi: 10.1371/journal.pone.0213952 (PMC7028272; doi:10.1371/journal.pone.0213952)
Supplement: S1 Table — MNI coordinates and number of voxels for each pre-defined ROI pair. (DOCX) [file pone.0213952.s002.docx]

**Supplementary Table S1.** MNI coordinates and number of voxels for each pre-defined ROI pair.

| **Tract name** | **“Seed” ROI** | | **“Target” ROI** | |
| --- | --- | --- | --- | --- |
|  | **Coordination**  **(x,y,z)** | **No. of 1mm^3^ Voxels** | **Coordination**  **(x,y,z)** | **No. of 1mm^3^ Voxels** |
| **Left MLF** | -2, -36, -50 | 1017 | -6, -26, -18 | 1664 |
| **Right MLF** | 4, -36, -50 | 628 | 8, -26, -18 | 1017 |
| **Left DLF** | -4, -38, -30 | 1095 | -2, -4, -10 | 1776 |
| **Right DLF** | 6, -38, -30 | 988 | 4, -4 -10 | 1461 |
| **Left SCP** | -6, -46, -28 | 1736 | -6, -24, -6 | 6585 |
| **Right SCP** | 8, -46, -28 | 1594 | 8, -24, -6 | 6562 |
| **Left NST** | -6, -20, -16 | 660 | -18, -4, -8 | 2339 |
| **Right NST** | 8, -20, -16 | 597 | 20, -2, -8 | 2265 |
| **Left MFT** | -8, 30, -24 | 4622 | -22, 39, -4 | 46896 |
| **Right MFT** | 8, 30, -24 | 3748 | 20, 39, -4 | 47433 |
| **Left FPT** | -10, -16, -20 | 2020 | -24, 8, 18 | 92443 |
| **Right FPT** | 10, -16, -20 | 1739 | 26, 8, 18 | 90500 |
| **Left CST** | -9, -20, -26 | 2120 | -24, -20, 54 | 13432 |
| **Right CST** | 9, -20, -26 | 1691 | 24, -20, 54 | 13425 |
| **Left STT** | -8, -34, -34 | 3473 | -20, -34, 54 | 16089 |
| **Right STT** | 8, -34, -34 | 3206 | 20, -34, 54 | 14659 |
| **Left POTPT (POPT)** | -11, -22, -14 | 1184 | -22, -44, 42 | 58523 |
| **Right POTPT (POPT)** | 11, -22, -14 | 1077 | 22, -44, 42 | 62872 |
| **Left POTPT (TPT)** | -12, -26, -26 | 348 | -26, -19, -6 | 3975 |
| **Right POTPT (TPT)** | 12, -26, -26 | 325 | 26, -19, -6 | 3934 |
