# Supplementary material for: Diffusion tensor tractography of brainstem fibers and its application in pain
Source: PLoS One. 2020 Feb 18;15(2):e0213952. doi: 10.1371/journal.pone.0213952 (PMC7028272; doi:10.1371/journal.pone.0213952)
Supplement: S2 Table — The success rates of each manual and automated tractographic performance. (DOCX) [file pone.0213952.s003.docx]

**Supplementary Table S2.** The success rates of each manual and automated tractographic performance.

| Brainstem fibers | Success rate (%) based on manual tractography | Success rate (%) based on automated tractography |
| --- | --- | --- |
| MLF | 100 | 100 |
| DLF | 100 | 100 |
| SCP | 100 | 100 |
| NST | 92 | 91 |
| MFT | 93 | 85 |
| FPT | 100 | 94 |
| CST | 88 | 88 |
| STT | 100 | 97 |
| POTPT | 100 | 100 |
