# Supplementary material for: Diffusion tensor tractography of brainstem fibers and its application in pain
Source: PLoS One. 2020 Feb 18;15(2):e0213952. doi: 10.1371/journal.pone.0213952 (PMC7028272; doi:10.1371/journal.pone.0213952)
Supplement: S3 Table — The reproducibility between manual and automated tractography based on the FA and fiber density measurements, respectively. (DOCX) [file pone.0213952.s004.docx]

**Supplementary Table S3.** The reproducibility between manual and automated tractography based on the FA and fiber density measurements, respectively.

| Brainstem fibers | Reproducibility (%) of FA measurement | Reproducibility (%) of Fiber density measurement |
| --- | --- | --- |
| MLF | 84 | 75 |
| DLF | 92 | 96 |
| SCP | 90 | 98 |
| NST | 94 | 79 |
| MFT | 82 | 64 |
| FPT | 90 | 79 |
| CST | 84 | 97 |
| STT | 90 | 71 |
| POTPT | 82 | 75 |

measurement reliability = ICC between measurement from manual tractography and measurement from automated tractography
