# Supplementary material for: Diffusion tensor tractography of brainstem fibers and its application in pain
Source: PLoS One. 2020 Feb 18;15(2):e0213952. doi: 10.1371/journal.pone.0213952 (PMC7028272; doi:10.1371/journal.pone.0213952)
Supplement: S4 Table — Estimated mean (S.E.) FA, mean, radial and axial diffusivity (MD, RD and AD) change rate (%) associated with per increased pain scale, R- Squared, effect size and significance that estimated based on bootstrapped-linear regression test for each brainstem tract. (DOCX) [file pone.0213952.s005.docx]

**Supplementary Table S4.** Estimated mean (S.E.) FA, mean, radial and axial diffusivity (MD, RD and AD) change rate (%) associated with per increased pain scale, R- Squared, effect size and significance that estimated based on bootstrapped-linear regression test for each brainstem tract.

| **Tract name** |  | **Pain Right Now**  **(n=12)** | | |  | **Worst Pain Last Month**  **(n=17)** | | |
| --- | --- | --- | --- | --- | --- | --- | --- | --- |
|  | **Mean (S.E.) % FA change rate /scale increase** | **R-Squared** | **Effect-size** | ***p*-value** | **Mean (S.E.) % FA change rate /scale increase** | **R-Squared** | **Effect-size** | ***p*-value** |
| **MLF** | **-3.02 (1.04)** | **0.533** | **-1.75** | **0.036** | **-1.87 (0.71)** | **0.346** | **-1.32** | **0.018** |
| **DLF** | **-3.48 (1.30)** | **0.664** | **-1.61** | **0.046** | **-2.30 (0.75)** | **0.487** | **-1.53** | **0.020** |
| **SCP** | -7.32 (3.28) | 0.655 | -1.34 | 0.068 | -2.98 (1.47) | 0.318 | -1.02 | 0.053 |
| **NST** | -0.72 (1.50) | 0.503 | -0.15 | 0.712 | -2.47 (1.83) | 0.394 | -0.75 | 0.204 |
| **MFT** | 0.94 (0.62) | 0.349 | 1.07 | 0.162 | 0.30 (0.53) | 0.293 | 0.33 | 0.581 |
| **FPT** | -0.60 (0.82) | 0.095 | -0.47 | 0.487 | 0.35 (0.63) | 0.076 | 0.29 | 0.599 |
| **CST** | 0.21 (1.05) | 0.046 | 0.12 | 0.914 | 0.84 (0.95) | 0.242 | 0.51 | 0.575 |
| **STT** | -0.59 (0.66) | 0.388 | -0.53 | 0.902 | 0.01 (0.68) | 0.228 | 0.01 | 0.991 |
| **POTPT** | -0.79 (1.39) | 0.105 | -0.38 | 0.459 | -0.04 (1.12) | 0.226 | -0.02 | 0.973 |
| **Tract name** | **Mean (S.E.) % MD change rate /scale increase** | **R-Squared** | **Effect-size** | ***p*-value** | **Mean (S.E.) % MD change rate /scale increase** | **R-Squared** | **Effect-size** | ***p*-value** |
| **MLF** | 0.07 (0.97) | 0.277 | 0.05 | 0.916 | -0.38 (0.61) | 0.260 | -0.31 | 0.468 |
| **DLF** | 1.39 (1.29) | 0.120 | 0.65 | 0.370 | 1.48 (1.01) | 0.144 | 0.73 | 0.220 |
| **SCP** | -1.47 (0.20) | 0.302 | -1.74 | 0.261 | 0.02 (2.00) | 0.152 | 0.01 | 0.988 |
| **NST** | -2.35 (4.60) | 0.034 | -0.31 | 0.563 | 4.04 (3.43) | 0.106 | 0.63 | 0.419 |
| **MFT** | -0.72 (0.78) | 0.237 | -0.65 | 0.420 | -0.23 (0.57) | 0.023 | -0.23 | 0.704 |
| **FPT** | -0.75 (0.39) | 0.361 | -1.22 | 0.159 | -0.39 (0.34) | 0.095 | -0.60 | 0.279 |
| **CST** | -0.66 (0.45) | 0.233 | -0.92 | 0.226 | -0.59 (0.38) | 0.189 | -0.88 | 0.288 |
| **STT** | 0.36 (0.44) | 0.079 | 0.49 | 0.521 | 0.44 (0.30) | 0.022 | 0.02 | 0.913 |
| **POTPT** | -0.96 (1.70) | 0.044 | -0.36 | 0.362 | -0.83 (1.21) | 0.057 | -0.36 | 0.340 |
| **Tract name** | **Mean (S.E.) % RD change rate /scale increase** | **R-Squared** | **Effect-size** | ***p*-value** | **Mean (S.E.) % RD change rate /scale increase** | **R-Squared** | **Effect-size** | ***p*-value** |
| **MLF** | 1.82 (1.36) | 0.218 | 0.83 | 0.127 | 0.90 (0.90) | 0.142 | 0.45 | 0.246 |
| **DLF** | 1.99 (1.33) | 0.252 | 0.92 | 0.236 | 2.12 (1.06) | 0.220 | 0.99 | 0.091 |
| **SCP** | 0.26 (1.17) | 0.061 | 0.12 | 0.798 | 1.49 (2.23) | 0.061 | 0.35 | 0.482 |
| **NST** | 0.59 (0.99) | 0.334 | 0.42 | 0.602 | 1.59 (1.11) | 0.135 | 0.11 | 0.113 |
| **MFT** | -1.09 (0.91) | 0.204 | -0.84 | 0.355 | -0.32 (0.65) | 0.036 | -0.23 | 0.731 |
| **FPT** | -0.38 (0.76) | 0.024 | -0.28 | 0.686 | -0.53 (0.53) | 0.109 | -0.55 | 0.406 |
| **CST** | -0.79 (0.99) | 0.086 | -0.53 | 0.600 | -1.12 (0.74) | 0.223 | -0.79 | 0.427 |
| **STT** | 0.67 (0.67) | 0.272 | 0.73 | 0.299 | 0.17 (0.50) | 0.163 | 0.13 | 0.836 |
| **POTPT** | 0.01 (2.17) | 0.044 | 0.00 | 0.995 | 0.68 (1.19) | 0.027 | 0.30 | 0.557 |
| **Tract name** | **Mean (S.E.) % AD change rate /scale increase** | **R-Squared** | **Effect-size** | ***p*-value** | **Mean (S.E.) % AD change rate /scale increase** | **R-Squared** | **Effect-size** | ***p*-value** |
| **MLF** | **-1.77 (0.89)** | **0.576** | **-1.21** | **0.046** | **-1.49 (0.51)** | **0.534** | **-1.45** | **0.016** |
| **DLF** | 0.60 (1.36) | 0.044 | 0.26 | 0.766 | 0.68 (5.39) | 0.088 | 0.65 | 0.599 |
| **SCP** | -5.81 (2.05) | 0.746 | -1.71 | 0.070 | -2.08 (2.17) | 0.352 | -0.48 | 0.442 |
| **NST** | -2.96 (5.40) | 0.033 | -0.33 | 0.538 | 4.16 (4.11) | 0.079 | 0.54 | 0.441 |
| **MFT** | -0.28 (0.67) | 0.382 | -0.30 | 0.651 | -0.17 (0.50) | 0.176 | -0.20 | 0.708 |
| **FPT** | -1.11 (0.37) | 0.594 | -1.91 | 0.054 | -0.25 (0.42) | 0.048 | -0.30 | 0.591 |
| **CST** | -0.47 (0.54) | 0.230 | -0.55 | 0.628 | -0.12 (0.45) | 0.235 | -0.16 | 0.799 |
| **STT** | -0.01 (0.45) | 0.152 | -0.01 | 0.983 | -0.03 (0.36) | 0.114 | -0.05 | 0.941 |
| **POTPT** | -1.04 (0.75) | 0.694 | -0.88 | 0.372 | -0.28 (0.63) | 0.473 | -0.24 | 0.582 |
